# Semantic Disease Gene Embeddings (SmuDGE): phenotype-based disease gene prioritization without phenotypes

**DOI:** 10.1093/bioinformatics/bty559

**Published:** 2018-09-08

**Authors:** Mona Alshahrani, Robert Hoehndorf

**Affiliations:** Computer, Electrical and Mathematical Sciences and Engineering Division, Computational Bioscience Research Center, King Abdullah University of Science and Technology, Thuwal, Saudi Arabia

## Abstract

**Motivation:**

In the past years, several methods have been developed to incorporate information about phenotypes into computational disease gene prioritization methods. These methods commonly compute the similarity between a disease’s (or patient’s) phenotypes and a database of gene-to-phenotype associations to find the phenotypically most similar match. A key limitation of these methods is their reliance on knowledge about phenotypes associated with particular genes which is highly incomplete in humans as well as in many model organisms such as the mouse.

**Results:**

We developed SmuDGE, a method that uses feature learning to generate vector-based representations of phenotypes associated with an entity. SmuDGE can be used as a trainable semantic similarity measure to compare two sets of phenotypes (such as between a disease and gene, or a disease and patient). More importantly, SmuDGE can generate phenotype representations for entities that are only indirectly associated with phenotypes through an interaction network; for this purpose, SmuDGE exploits background knowledge in interaction networks comprised of multiple types of interactions. We demonstrate that SmuDGE can match or outperform semantic similarity in phenotype-based disease gene prioritization, and furthermore significantly extends the coverage of phenotype-based methods to all genes in a connected interaction network.

**Availability and implementation:**

https://github.com/bio-ontology-research-group/SmuDGE

## 1 Introduction

There is now a large number of available methods for the prioritization or prediction of gene–disease associations (Natarajan and Dhillon, 2014; Wang *et al.*, 2011; Zhou and Skolnick, 2016). Computational methods that predict gene–disease associations use a large number of different features and approaches.

Several approaches to the computational prediction of gene–disease associations are based on the guilt-by-association principle (Gillis and Pavlidis, 2012). Using the guilt-by-association approach relies on prior knowledge of a set of genes associated with a disease *D* and a relatedness measure that compares genes with the set of genes associated with *D*; if a gene is strongly related with respect to the relatedness measure it is suggested as a novel candidate gene. Several measures are used to determine relatedness between genes, with the most prominent ones relying on network associations (Aerts, 2006; Köhler *et al.*, 2008; Lee *et al.*, 2011) or some form of functional or phenotypic similarity (Schlicker and Albrecht, 2008). However, as guilt-by-association relies on prior knowledge of disease-associated genes, they cannot easily be applied to monogenic diseases, and their applications are, in general, limited to few diseases.

Phenotype-based approaches have been particularly successful in finding candidate genes for Mendelian diseases (Hoehndorf *et al.*, 2011). Phenotype-based approaches compare disease phenotypes to a database of genotype–phenotype associations and suggest candidate genes based on measures of phenotype similarity (Eilbeck *et al.*, 2017; Hoehndorf *et al.*, 2011; Köhler *et al.*, 2009).

The main limitation of phenotype-based approaches, however, is the limited amount of phenotype annotations that are associated with particular genotypes in public databases. In the past, one approach to address this limitation is the use of phenotype associations resulting from animal model experiments and the use of ontologies that can combine phenotypes across species so that animal model and human phenotypes can be compared (Chen *et al.*, 2012; Gkoutos *et al.*, 2017; Hoehndorf *et al.*, 2011). While the use of model organisms significantly extends the scope of phenotype-based disease-gene prioritization methods, there is nevertheless only a limited amount of phenotype associations available. In particular, genes for which there are no orthologs in other organisms cannot benefit from cross-species phenotype-based approaches. One possible way in which this challenge could be overcome is to predict phenotypes associated with genes that have no such associations in databases, for example through the use of background knowledge in the form of interaction networks.

We developed SmuDGE, a method that generates features encoding phenotype-associations, interaction network connectivity patterns for any gene in the interaction network. These features can be used to predict gene–disease associations. To achieve this goal, SmuDGE combines phenotype similarity with network-based representation learning and propagates information about phenotype-associations through interaction network connections. We demonstrate that SmuDGE can be used to identify candidate genes of disease through the use of phenotype similarity even if no phenotypes are associated with a gene. SmuDGE is freely available from https://github.com/bio-ontology-research-group/SMUDGE.

## 2 Materials and methods

### 2.1 Data sources and versions

We use the PhenomeNET ontology (Rodríguez-García *et al.*, 2017), downloaded on 8 Jun 2018 from the AberOWL repository (Hoehndorf *et al.*, 2015), as our phenotype ontology because it integrates human and model organism phenotypes and allows them to be compared. We use a dataset of diseases with their phenotypes from the HPO database (Köhler *et al.*, 2014), downloaded on 8 Jun 2018.

Furthermore, we use gene-to-phenotype associations observed in mutant mouse models, downloaded from the Mouse Genome Informatics (MGI) database (Blake *et al.*, 2014) on 8 Jun 2018, and gene-to-phenotype associations derived from gene–disease associations and provided by the HPO database, downloaded on 8 Jun 2018. We further used the interactions provided by STRING (Szklarczyk *et al.*, 2011) version 10. STRING contains both direct and indirect interactions.

Our dataset consists of 7149 OMIM diseases with 78 402 associations to 6596 distinct phenotypes; 3526 human genes with 153 575 associations to 6058 distinct phenotypes; and 12 037 mouse genes with 209 387 associations to 9292 distinct phenotypes. We use human–mouse orthology obtained from MGI on 8 Jun 2018 to identify the human orthologs of mouse genes, and associate mouse gene phenotypes with their human orthologs, resulting in 152 159 associations between 9482 human genes and 9215 distinct phenotypes.

Furthermore, we map all proteins in the STRING interaction network to their gene identifiers using the mappings provided by STRING. The resulting interaction network between genes consists of 493 041 interactions between 14 753 genes.

For evaluation, we used 12 469 gene–disease associations for 3159 OMIM diseases, found in the file (MGI_DO.rpt) at MGI. We use only the gene–disease associations in humans from this file.

### 2.2 Construction of the heterogeneous graphs

We have built two kinds of heterogeneous knowledge graphs to study gene–disease associations. The first knowledge graph utilizes the cross-species PhenomeNET ontology (Rodríguez-García *et al.*, 2017) and characterizes phenotypes of human diseases and mouse models. We associate the human orthologs of the mouse genes with mouse phenotypes, resulting in 152 159 associations between human genes and mouse phenotypes. Furthermore, we construct a second version of that graph in which we use human proteins and assign them with their phenotypes obtained from the HPO database.

The second graph aims to exploit a protein-protein interaction network to generate vector representations for genes which don’t have phenotypes. It consists of the same information as the first type of graph plus the STRING interaction network (Szklarczyk *et al.*, 2011).

### 2.3 Similarity computation and evaluation

We use cosine similarity between two vectors *v*_1_ and *v*_2_ to determine the similarity of embeddings:

$$sim(v_{1},v_{2})=\frac{v_{1}\cdot v_{2}}{\left||v_{1}||\;||v_{2}|\right|}$$

We use cosine similarity to compute the similarity between disease and gene embeddings. We use their similarity as predictor for a gene being associated with a disease.

As a baseline for comparison, we use a semantic similarity measure which exploits the background knowledge in an ontology. We use Resnik’s semantic similarity measure (Resnik *et al.*, 1999) with the Best Match Average (BMA) strategy for combining similarities between individual classes. Resnik’s semantic similarity measure is defined as:

(1) $$Sim(c_{1},c_{2})=\underset{c\in S(c_{1},c_{2})}{\max}[-\text{log}\;p(c)]$$

where *c*_1_ and *c*_2_ are the two classes between which similarity is computed, and $S(c_{1},c_{2})$ is the set of superclasses of both *c*_1_ and *c*_2_ in the ontology hierarchy and *p*(*c*) is the probability of a disease or gene being associated with class *c*.

We also compare SmuDGE results with the simGIC semantic similarity measure (Pesquita *et al.*, 2008). simGIC is defined as:

(2) $$simGIC(c_{1},c_{2})=\frac{\Sigma_{c\in S(c_{1})\cap S(c_{2})}-\text{log}\;p(c)}{\Sigma_{c\in S(c_{1})\cup S(c_{2})}-\text{log}\;p(c)}$$

To evaluate the performance of the similarity-based predictions, we compute a similarity matrix which contains the pairwise similarities of genes and diseases. For each disease, we rank genes in descending order of the similarity score. We then evaluate at which rank we identify a gene–disease association in our evaluation dataset. As this method results in a ranking classifier (as genes are ranked for each disease), we quantify the performance of the predictions through the area under the receiver operating characteristic (ROC) curve (Fawcett, 2006). A ROC curve is a plot of the true positive rate (TPR) as a function of the false positive rate (FPR). The 

TPR at a particular rank is defined as a rate of correctly predicted gene–disease associations at this rank, and the FPR is the rate of predicted associations that are not gene–disease associations. As we do not have true negative gene–disease associations, we treat unknown gene–disease associations as negatives.

### 2.4 Supervised prediction and evaluation

SmuDGE is an unsupervised method to generate feature vectors for genes and diseases based on their phenotypes. Using these features in a supervised manner can improve the prediction of associations between two vectors in comparison to use of a pre-defined similarity measure (Smaili *et al.*, 2018). For this reason, we use an artificial neural network (ANN) and train it to predict gene–disease associations from embedding vectors.

In this experiment, we use the known disease-gene associations as the positive set, and randomly select an equal number of the non-associated disease-gene pairs as the negative set.

For the the training and testing, we perform 5-fold cross validation. We generate folds by sampling diseases, not gene–disease pairs. 80% of the diseases are used for training the ANN and 20% of the diseases for testing. As positive pairs, we combine the disease embeddings in each fold with the gene embeddings for genes associated with the diseases. The aim of this sampling strategy is to guarantee that the ANN does not learn to recognize gene–disease associations for a disease *D* based on genes known to be associated with *D*, and therefore determine how well our method predicts genes associated with diseases if no prior knowledge is available.

We used 10% of the training set as a validation set to guide and stop the training if the loss increases in the validation set; alternatively, training will stop after 100 epochs. We use a Rectified Linear Unit as an activation function for the hidden layers (Nair and Hinton, 2010) and a sigmoid function as the activation function for the output layer; we use cross entropy as loss function in training, and Rmsprop (Hinton *et al.*, 2012) to optimize the neural networks parameters during training.

For the evaluation of the ROCAUC, we create an embedding matrix for each disease in which we fix the first part of the matrix to represent a particular disease embeddings and the second part represents the all gene embeddings. We then apply our model and rank genes based on the model’s prediction scores. The TPR and FPR at each rank are used to identify the proportion of correctly and falsely predicted associations.

## 3 Results

### 3.1 Heterogeneous representation of genes, diseases and phenotypes

In our method we use a knowledge graph as data structure in which we represent genes, diseases, and the phenotypes with which they are associated. Genes, diseases, and phenotypes are represented as nodes in the graph. Edges between phenotypes represent axioms in the Web Ontology Language (OWL) (Grau *et al.*, 2008; Rodríguez-García and Hoehndorf, 2018). We represent diseases using their identifiers from the Online Mendelian Inheritance in Men (OMIM) (Amberger *et al.*, 2011)) database, human genes using their Entrez gene identifier, and phenotypes using the cross-species phenotype ontology PhenomeNET (Rodríguez-García *et al.*, 2017). We connect diseases and genes to the phenotypes they are associated with using the *has phenotype* relation. Additionally, we represent interactions between genes and their products using an *interacts with* relation. We consider all *interacts with* edges as symmetric (i.e. it *x* interacts-with *y* then *y* interacts-with *x*) and all other edges as non-symmetric.

We associate all OMIM diseases with their phenotypes from the Human Phenotype Ontology (HPO) database (Köhler *et al.*, 2017), and obtain information about interactions between human genes from the STRING database (Szklarczyk *et al.*, 2011).

We build two knowledge graphs which differ in the associations between genes and their phenotypes. In the first case, we use the phenotypes associated with human genes in the HPO database; these phenotypes are indirectly derived from gene–disease associations and the disease phenotypes, i.e. if a gene *G* is associated with a disease *D* and the disease has a set of phenotypes *P*, then all phenotypes in *P* are assigned to *G*. Because this assignment of phenotypes to human genes can indirectly encode gene–disease associations, we also use mouse model phenotypes as an independent dataset of phenotypes. For this purpose, we identify the phenotypes of non-conditional loss of function mutations (i.e. knockouts) of gene *G* in the Mouse Genome Informatics (MGI) database (Blake *et al.*, 2014); if we can identify a human ortholog of *G*, we assign all phenotypes of *G* to the human ortholog. Phenotypes of mouse models are encoded using the Mammalian Phenotype Ontology (MP) (Smith *et al.*, 2004); through the use of the cross-species PhenomeNET ontology (Rodríguez-García *et al.*, 2017), the phenotypes encoded using MP (in the mouse) and HPO (in human disease) can be compared directly.

**Fig. 1.**
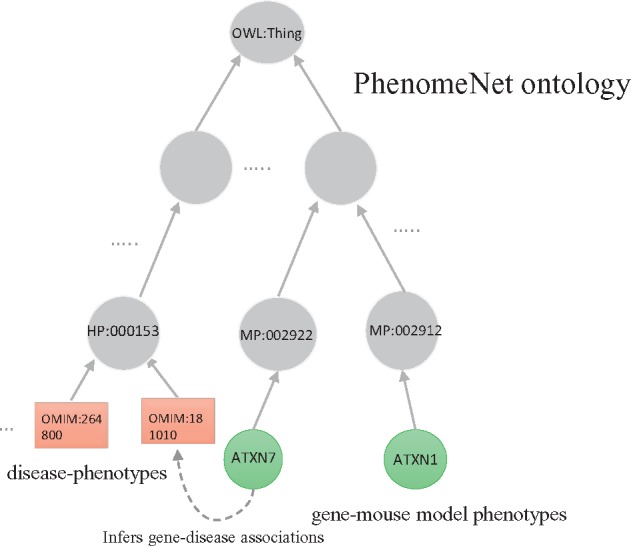
Our knowledge graph consists of gene–phenotype associations (encoded using either HPO or MP), disease–phenotype associations (encoded using the HPO), interactions between genes (from the STRING database) and the PhenomeNET ontology

Our graphs consist of 7149 nodes for OMIM diseases and 15 391 nodes for human genes. Including the PhenomeNET ontology, it further contains 12 289 nodes for MP classes and 12 007 nodes for HPO classes. It has 78 599 disease–phenotype associations. Using phenotypes from HPO, we include 153 575 gene–phenotype associations; using mouse phenotypes from MGI, we include 152 159 gene–phenotype associations. We also include 493 041 interactions between genes, all of which we consider as symmetric. Figure 1 illustrates the knowledge graph we generate.

### 3.2 Joint representation learning from PPI network structure and phenotype annotations

**Fig. 2.**
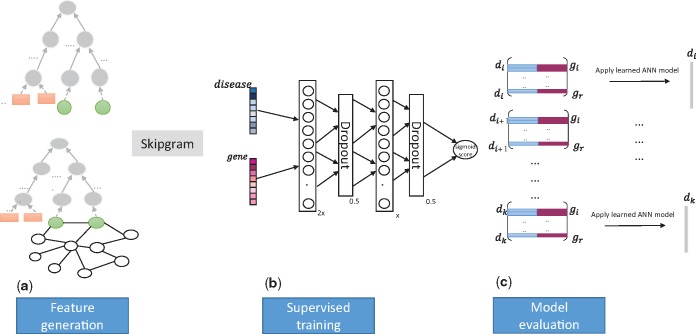
Overview over SmuDGE and its applications. (**a**) On the left side we show the graph of disease–phenotype and gene–phenotype associations together with the PhenomeNET ontology (top), and the same graph including interactions between genes used to generate E-Vecs at the bottom. We generate a corpus by graph traversal and then use a skipgram model to generate vectors for genes and gene products in the graph. These vectors can be used as input to a similarity measure, or a neural network, to predict interactions between genes and diseases. (**b**) Our ANN model is shown in the center; the input is the pair of disease and gene feature vectors of dimension *x*, the first hidden layer consists of 2*x* hidden units, and the second hidden layer consist of *x* hidden units; we use a dropout of 0.5 to mitigate the effects of overfitting. (**c**) We evaluate the model by predicting candidate genes for each disease and rank each gene for each disease based on the ANN’s prediction score

We designed an algorithm to encode features based on the phenotypes that are associated with entities in the knowledge, either diseases or genes and gene products, in the form of a dense vector; the vector representation of the genes can then be used in unsupervised or supervised machine learning approaches or other predictive models. Figure 2 provides a high-level overview over our algorithm.

Our algorithm, Semantic Disease Gene Embeddings (SmuDGE), comes in two forms. First, it encodes the phenotypes that are directly associated with an entity (i.e. a disease or gene/gene product); for this purpose, it generates a dense representation of an entities ontology-based annotations and its superclasses. This algorithm is applicable to all diseases and genes that are directly associated with phenotypes. However, while diseases are commonly associated with (or even defined by) a set of phenotypes, the majority of genes are not associated with phenotypes, neither in humans, where phenotypes are generally derived from gene–disease associations (Köhler *et al.*, 2014), nor in the mouse where phenotypes are the result of phenotyping experiments (de Angelis *et al.*, 2015). Therefore, we use a second form of our algorithm which is applicable to genes without any phenotype associations and in which a phenotype-based representation is assigned indirectly using the network environment in which a gene product is embedded.

For the first version of our algorithm, we ignore all interactions between genes and their products and focus only on encoding an entity’s phenotype annotations and the ontology structure (i.e. subclass relations). Given an entity *E* (disease or gene) that has *has phenotype* edges to the phenotypes $P_{1},\ldots ,P_{n}$, we generate *n* sentences starting with *E*. We generate *n* sentences where each sentence starts with *E* followed by *P_i_* and all of the superclasses of *P_i_*, for all *P_i_* that are directly associated with *E*.

We then apply a Word2Vec skipgram model (Mikolov *et al.*, 2013) to learn vector representations for each token occurring in a generated sentence, in particular for all entities and phenotype classes. The vectors generated for entities through this approach encode directly associated phenotypes and all their superclasses, and we call the vectors *P-Vecs* (for Phenotype Vectors).

We can only generate P-Vecs for genes and gene products with directly associated phenotypes. For all other genes, however, we can use their interaction network environment to assign phenotypes that are over-represented in the neighboring nodes. Similar to generating knowledge graph embeddings (Alshahrani *et al.*, 2017), for a gene *G*, we use a random walk, starting at *G*, over the network of interactions between genes and gene products to randomly sample *G*’s network neighborhood. We terminate the walk once we found a node G′ with *has phenotype* edges, or after a pre-determined step limit, whichever occurs first. If the step limit has been reached, we restart the walk at *G*. If the walk found a G′ with an outgoing *has phenotype* edge, and the phenotypes associated with G′ are $P_{1},\ldots ,P_{m}$, then we randomly sample one phenotype *P* of $P_{1},\ldots ,P_{m}$ and generate a sentence starting with *G* followed by *P* and all superclasses of *P*; after adding the sentence to our corpus, we restart at *G* until a maximum number of walks is reached. The aim of this approach is to sample the network environment in which *G* is located for phenotypes. Through inclusion of the ontology hierarchy in the generated sentences, the approach is intended to be more robust to differences in specific phenotypes. Similarly to generating P-Vecs, we apply a Word2Vec skipgram model on the generated sentences to produce vector representations of all entities and phenotypes in the corpus. Because these representations are generated from a gene node’s network environment, we call the vectors *E-Vecs* (for Environment Vectors). Figure 2 provides a high-level overview over our method.

We generate both P-Vecs and E-Vecs for our two graphs (using human and mouse gene–phenotype associations separately). We generate P-Vecs for all human diseases, and we further generate P-Vecs both for genes that have human and mouse phenotypes associated. We generate E-Vecs both for nodes which do not have phenotypes associated and for nodes which have phenotypes associated; if a gene node has directly associated phenotypes, we mask them during the generation of sentences (i.e. random walks) for the E-Vec approach so that only the network environment is sampled for phenotypes. Furthermore, we generate vector representations for all phenotype classes from HPO and MP which are either used to directly annotate a gene or diseases, or are a superclass of a direct annotation.

In total, we generate 12 289 embedding vectors for phenotype classes from MP, 12 007 for phenotype classes from HPO, 7150 for diseases from OMIM, 9482 P-Vecs for genes using mouse phenotypes (i.e. assigning phenotypes of mouse genes to their human orthologs), and 3526 P-Vecs for genes using human phenotype data (i.e. genes-phenotypes associations provided by the HPO database). We generate E-Vecs for 14 753 genes, i.e. for all genes in our interaction network that are connected to a gene with phenotype associations.

### 3.3 Similarity-based prediction of disease-associated genes

We use the generated vectors representing genes and diseases to predict gene–disease associations based on phenotype similarity. The SmuDGE vectors encode phenotype annotations connectivity patterns along with the ontology super classes associated with each phenotype annotation. Similar phenotypes for both genes and diseases indicate similar features vectors and therefore we can infer disease-gene associations by comparing feature vectors.

**Fig. 3.**
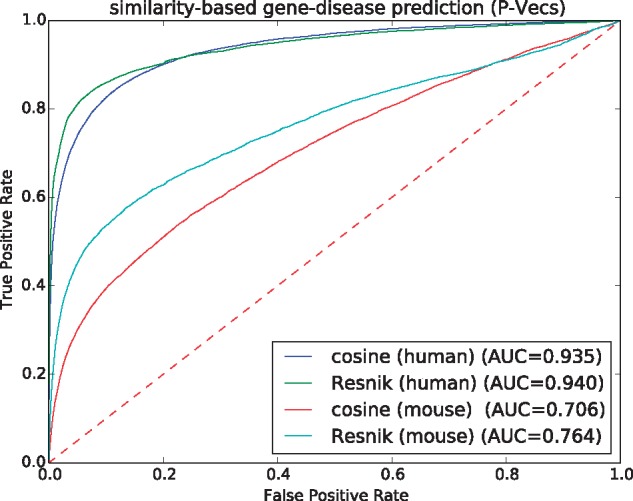
ROC curves for predicting gene–disease associations using cosine similarity between SmuDGE’s P-Vecs and comparison to Resnik’s semantic similarity measure

To determine similarity of the resulting features vectors, we use the cosine similarity measure and we compute the pairwise similarity between all OMIM diseases and genes (using either their P-Vec or E-Vec representation). We then rank the most similar genes for each disease and determine how well we recover known gene–disease associations from OMIM using a receiver operating characteristic (ROC) curve (Fawcett, 2006); we quantify the performance of the similarity-based prediction using the area under the ROC curve (ROCAUC) which is equivalent to the probability that a randomly chosen positive sample is ranked higher than a randomly chosen negative sample. We limit our evaluation of P-Vec similarity to the genes for which we can generate the representations, i.e. 3526 genes using human phenotypes and 9482 genes using mouse phenotypes. For comparison, we use a semantic similarity measure to compare disease and gene phenotype annotations. For the evaluation of E-Vec similarity, since we generate representations for all of the genes in the interactions network, we evaluate the disease vectors against the set of 14 753 genes vectors. Figure 3 shows the results using P-Vec similarity for human and mouse phenotypes. Table 1 shows a summary of applying both approaches (i.e. P-Vec and E-Vec) using human and mouse phenotypes.

**Table 1.** Summary of the ROCAUCs for predicting gene–disease associations using human and mouse phenotypes and using the P-Vec and E-Vec approaches

Approach	P-Vec	P-Vec	E-Vec	E-Vec
phenotype source	human	mouse	human	Mouse
SmuDGE (cosine)	0.935	0.706	0.765	0.673
SmuDGE (ANN)	0.972	0.911	0.871	0.839
Resnik	0.940	0.764	N/A	N/A
simGIC	0.858	0.713	N/A	N/A

*Note***:** We compare the results to Resnik and simGIC semantic similarity; these measures are ontology-based semantic similarity measures and only comparable to SmuDGE’s P-Vec approach as genes without phenotype annotations cannot benefit from semantic similarity.

We find that P-Vec similarity using human phenotypes results in almost perfect prediction, which is the consequence of how the phenotypes have been assigned to genes in the HPO database (i.e. the phenotypes are identical to the phenotypes of the disease with which the gene is associated, and using them for prediction is therefore almost circular); these similarities are therefore not truly predictive but mainly reproduce our evaluation dataset. However, using mouse phenotypes, we obtain a ROCAUC of 0.706 when comparing P-Vecs to the disease vectors. Using E-Vecs, we obtain a ROCAUC of 0.765 when using human gene–phenotype associations and a ROCAUC of 0.673 using gene–phenotype associations from the mouse. Notably, because we mask all direct gene–phenotype associations when generating E-Vecs, our use of human gene–phenotype associations does not encode gene–disease associations, and the performance of this similarity-based evaluation is therefore indicative of predicting disease-associated genes in the absence of gene–phenotype associations.

### 3.4 Supervised prediction of disease-associated genes

Cosine similarity can only be applied to vectors of the same dimension, and furthermore cannot easily account for dataset-specific features. Therefore, we also apply supervised machine learning to ‘learn’ a function (akin to a similarity measure) that takes two phenotype-based representation vectors as input and is predictive of gene–disease associations. We use an artificial neural network (ANNs) to learn these functions in a supervised manner; the ANN model accepts a pair of features vectors (i.e. embeddings) $z_{d}\in R^{d}$ and $z_{g}\in R^{d}$ corresponding to entities *E_d_*, *E_g_* for disease and gene nodes.

Several approaches to computational prediction of gene–disease associations utilize the principle known as ‘guilt-by association’ (Gillis and Pavlidis, 2012) which infers the associations of a gene to a disease based on the similarity to other genes associated with the disease. As a result, it fails to predict genes for diseases with no prior knowledge of any associated genes. Supervised training to predict gene–disease associations is similar to the guilt-by-association approach if some genes associated with a disease have been used in training and the model is evaluated on the remaining genes, because knowledge about disease-associated genes is used to predict more associations. To estimate the performance of our method for predicting gene-associations for diseases without associated genes,

We first split the training and testing based on diseases, not on gene–disease pairs. In particular, we select 80% of the diseases and all their associated genes for training, and apply the model to predict all the genes for the remaining 20% of the diseases and their associated genes for testing.

**Fig. 4.**
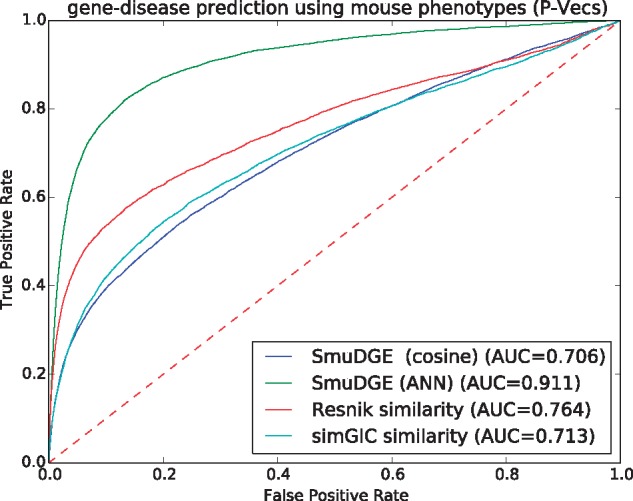
Comparision of ROC curves for predicting gene–disease associations based on mouse phenotypes using SmuDGE’s feature vectors and comparison to the Resnik and simGIC semantic similarity measures

We evaluate each type of vector representation individually using our ANN model approach (see Section 2). As in the similarity-based prediction of disease-associated genes, we use pairs of P-Vecs or E-Vecs as input to the ANN and use the ANN to compute the ‘similarity’ between them as a predictor of gene–disease associations. The ROC curves for using mouse phenotypes, as well as the comparison to other semantic similarity measures, are shown in Figure 4 and Table 1. We find that using the ANN significantly improves the results compared to the unsupervised, similarity-based approach, increasing the ROCAUC from 0.935 to 0.972 for human phenotypes and from 0.706 to 0.911 for mouse phenotypes with P-Vec approach as well as using E-Vecs from 0.764 to 0.871 with human phenotypes and from 0.673 to 0.839.

**Fig. 5.**
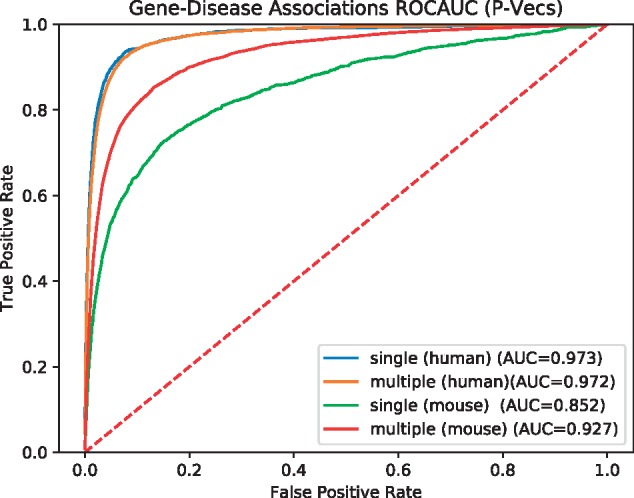
ROC curves for predicting gene–disease associations for diseases with a single or multiple associated genes using SmuDGE’s P-Vec approach

**Fig. 6.**
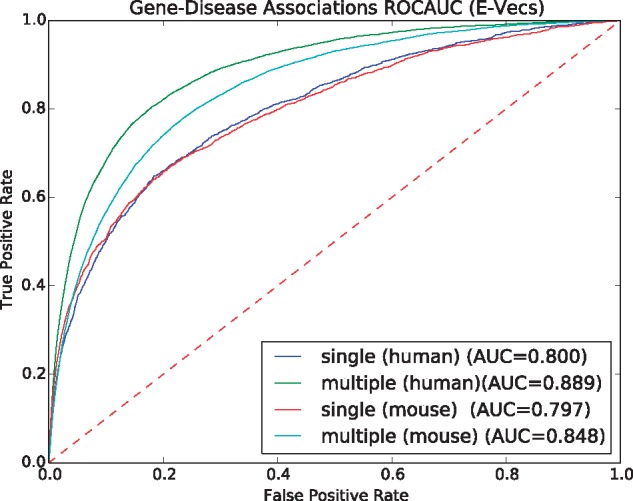
ROC cuves for predicting gene–disease associations for diseases with a single or multiple associated genes using SmuDGE’s E-Vec approach

As another use case, we also evaluated how well SmuDGE can predict gene–disease associations for diseases with only a single association compared to diseases with multiple associated genes. Although our dataset is stratified by disease and known associations are therefore not used during training of the neural network, we intend to test the performance of our approach on rare diseases for which no or only little information is available. Figures 5 and 6 show the result. We find that for diseases which have more gene associations and are likely better studied, SmuDGE can predict associated genes better than for diseases with only a single associated gene, using both the P-Vec and E-Vec approach.

## 4 Discussion

SmuDGE is an algorithm that exploits ontologies and knowledge graphs to learn representations of genes, gene products and diseases, based on the phenotypes they are associated with. While we demonstrate in our evaluation that the performance of SmuDGE in predicting gene–disease associations matches, and sometimes outperforms, traditional phenotypes-based gene prioritization methods such as PhenomeNET (Hoehndorf *et al.*, 2011) or the MouseFinder (Chen *et al.*, 2012), we see our main contribution in extending the phenotype- and similarity-based approaches for gene–disease prioritization to all genes represented in an interaction network (or knowledge graph).

The prediction of disease genes using phenotype-similarity has been highly successful (Chen *et al.*, 2012; Hoehndorf *et al.*, 2011; Köhler *et al.*, 2009) and a major limitation has been the availability of phenotypes for many genes. The use of non-human model organisms such as the mouse (Meehan *et al.*, 2017) can generate phenotype representations of human genes even in the absence of clinically determined phenotypes associated with a gene; however, even using model organism phenotypes, phenotype similarity can still not be applied to a large portion of human genes due to missing data or lack of a non-human ortholog.

SmuDGE’s E-Vecs don’t use directly associated phenotypes but encode phenotypes of a gene based on knowledge of interactions—both direct and indirect—of a gene with other genes with which phenotypes are associated. The disease vector representations we generate always encode phenotypes, and the success in identifying gene–disease associations when comparing both demonstrates that E-Vecs and disease phenotype vectors have similar (and possibly complementary) information, sufficient for their comparison to be predictive of disease mechanisms (i.e. disease-associated genes).

The E-Vecs we construct in our work further encode, although indirectly, for phenotypic network modules, since they are generated through random walks on an interaction network and will encode phenotypes overrepresented within a network region. In future work, we plan to evaluate whether our approach can identify interacting genes that may be jointly associated with a disease, such as in digenic and other oligogenic diseases (Gazzo *et al.*, 2016). We may also explore the possibility to combine SmuDGE with variant prioritization tools to provide additional information that can be used to determine whether a variant is associated with a phenotype or not (Boudellioua *et al.*, 2017).

## 5 Conclusions

SmuDGE is a method to generate semantic disease gene embeddings and use them to predict gene–disease associations. SmuDGE is phenotype-based and can be used to predict disease-associated genes by computing the similarity between the phenotypes associated with a disease and those associated with a gene; utilizing an interaction network as background knowledge and assigning phenotype-based representations to genes that have no associated phenotypes, SmuDGE is applicable to any gene for which either phenotype associations or background knowledge about interactions with other genes that have phenotype associations is available. We have demonstrated through multiple experiments that SmuDGE can improve the state of the art in phenotype-based prioritization of disease genes. We envision the main application of SmuDGE in the prioritization of genes for rare genetic diseases.

## Funding

This work was supported by funding from King Abdullah University of Science and Technology (KAUST) Office of Sponsored Research (OSR) under Award No. URF/1/3454-01-01 and FCC/1/1976-08-01.

*Conflict of Interest*: none declared.

## References

[bty559-B1] Aerts S. (2006) Gene prioritization through genomic data fusion. Nat. Biotechnol., 24, 537–544. 16680138 10.1038/nbt1203

[bty559-B2] Alshahrani M. et al (2017) Neuro-symbolic representation learning on biological knowledge graphs. Bioinformatics, 33, 2723–2730. 28449114 10.1093/bioinformatics/btx275 PMC5860058

[bty559-B3] Amberger J. et al (2011) A new face and new challenges for Online Mendelian Inheritance in Man (OMIM). Hum. Mutat., 32, 564–567. 21472891 10.1002/humu.21466

[bty559-B4] Blake J.A. et al (2014) The mouse genome database: integration of and access to knowledge about the laboratory mouse. Nucleic Acids Res., 42, D810–D817. DOI: 10.1093/nar/gkt1225. 24285300 10.1093/nar/gkt1225 PMC3964950

[bty559-B5] Boudellioua I. et al (2017) Semantic prioritization of novel causative genomic variants. PLoS Comput. Biol., 13, e1005500. 28414800 10.1371/journal.pcbi.1005500 PMC5411092

[bty559-B6] Chen C.-K. et al (2012) Mousefinder: candidate disease genes from mouse phenotype data. Hum. Mutat., 33, 858–866. 22331800 10.1002/humu.22051 PMC3327758

[bty559-B7] de Angelis M.H. et al (2015) Analysis of mammalian gene function through broad-based phenotypic screens across a consortium of mouse clinics. Nat. Genet., 47, 969–978. 10.1038/ng.3360 PMC4564951 26214591

[bty559-B8] Eilbeck K. et al (2017) Settling the score: variant prioritization and mendelian disease. Nat. Rev. Genet., 18, 599–612. 28804138 10.1038/nrg.2017.52 PMC5935497

[bty559-B9] Fawcett T. (2006) An introduction to ROC analysis. Pattern Recogn. Lett., 27, 861–874.

[bty559-B10] Gazzo A.M. et al (2016) Dida: a curated and annotated digenic diseases database. Nucleic Acids Res., 44, D900. 26481352 10.1093/nar/gkv1068 PMC4702791

[bty559-B11] Gillis J., Pavlidis P. (2012) ‘Guilt by Association’ is the exception rather than the rule in gene networks. PLoS Comput. Biol., 8, e1002444. 22479173 10.1371/journal.pcbi.1002444 PMC3315453

[bty559-B12] Gkoutos G.V. et al (2017) The anatomy of phenotype ontologies: principles, properties and applications. Brief. Bioinf., in press. 10.1093/bib/bbx035 PMC6169674 28387809

[bty559-B13] Grau B. et al (2008) OWL 2: the next step for OWL. Web Semantics Sci. Services Agents World Wide Web, 6, 309–322.

[bty559-B14] Hinton G. et al (2012) Neural networks for machine learning lecture 6a overview of mini-batch gradient descent. https://www.cs.toronto.edu/∼tijmen/csc321/slides/lecture_slides_lec6.pdf (19 July 2018, date last accessed).

[bty559-B15] Hoehndorf R. et al (2011) Phenomenet: a whole-phenome approach to disease gene discovery. Nucleic Acids Res., 39, e119. 21737429 10.1093/nar/gkr538 PMC3185433

[bty559-B16] Hoehndorf R. et al (2015) Aber-OWL: a framework for ontology-based data access in biology. BMC Bioinformatics, 16, 26. 25627673 10.1186/s12859-015-0456-9 PMC4384359

[bty559-B17] Köhler S. et al (2008) Walking the interactome for prioritization of candidate disease genes. Am. J. Hum. Genet., 82, 949–958. 18371930 10.1016/j.ajhg.2008.02.013 PMC2427257

[bty559-B18] Köhler S. et al (2014) The human phenotype ontology project: linking molecular biology and disease through phenotype data. Nucleic Acids Res., 42, D966–D974. 24217912 10.1093/nar/gkt1026 PMC3965098

[bty559-B19] Köhler S. et al (2009) Clinical diagnostics in human genetics with semantic similarity searches in ontologies. Am. J. Hum. Genet., 85, 457–464. 19800049 10.1016/j.ajhg.2009.09.003 PMC2756558

[bty559-B20] Köhler S. et al (2017) The human phenotype ontology in 2017. Nucleic Acids Res., 45, D865–D876. 27899602 10.1093/nar/gkw1039 PMC5210535

[bty559-B21] Lee I. et al (2011) Prioritizing candidate disease genes by network-based boosting of genome-wide association data. Genome Res., 21, 1109–1121. 21536720 10.1101/gr.118992.110 PMC3129253

[bty559-B22] Meehan T.F. et al (2017) Disease model discovery from 3, 328 gene knockouts by the international mouse phenotyping consortium. Nat. Genet., 49, 1231–1238. 28650483 10.1038/ng.3901 PMC5546242

[bty559-B23] Mikolov T. et al (2013) Distributed representations of words and phrases and their compositionality. In: *Advances in Neural Information Processing Systems*, pp. 3111–3119.

[bty559-B24] Nair V., Hinton G.E. (2010). Rectified linear units improve restricted boltzmann machines. In: *Proceedings of the 27th International Conference on Machine Learning (ICML-10)*, pp. 807–814.

[bty559-B25] Natarajan N., Dhillon I.S. (2014) Inductive matrix completion for predicting genedisease associations. Bioinformatics, 30, i60–i68. 24932006 10.1093/bioinformatics/btu269 PMC4058925

[bty559-B26] Pesquita C. et al (2008) Metrics for GO based protein semantic similarity: a systematic evaluation. BMC Bioinformatics, 9, S4. 10.1186/1471-2105-9-S5-S4 PMC2367622 18460186

[bty559-B27] Resnik P. et al (1999) Semantic similarity in a taxonomy: an information-based measure and its application to problems of ambiguity in natural language. J. Artif. Intell. Res. (JAIR), 11, 95–130.

[bty559-B28] Rodríguez-García M.Á., Hoehndorf R. (2018) Inferring ontology graph structures using owl reasoning. BMC Bioinformatics, 19, 7. 29304741 10.1186/s12859-017-1999-8 PMC5756413

[bty559-B29] Rodríguez-García M.Á. et al (2017) Integrating phenotype ontologies with phenomenet. J. Biomed. Semantics, 8, 58. 29258588 10.1186/s13326-017-0167-4 PMC5735523

[bty559-B30] Schlicker A., Albrecht M. (2008) Funsimmat update: new features for exploring functional similarity. Nucleic Acids Res., 36, D434–D439. 17932054 10.1093/nar/gkm806 PMC2238903

[bty559-B31] Smaili F.Z. et al (2018) Onto2vec: joint vector-based representation of biological entities and their ontology-based annotations. *Bioinformatics*, 34, i52–i60. 10.1093/bioinformatics/bty259 PMC6022543 29949999

[bty559-B32] Smith C.L. et al (2004) The mammalian phenotype ontology as a tool for annotating, analyzing and comparing phenotypic information. Genome Biol., 6, R7. 15642099 10.1186/gb-2004-6-1-r7 PMC549068

[bty559-B33] Szklarczyk D. et al (2011) The STRING database in 2011: functional interaction networks of proteins, globally integrated and scored. Nucleic Acids Res., 39, D561–D568. 21045058 10.1093/nar/gkq973 PMC3013807

[bty559-B34] Wang X. et al (2011) Network-based methods for human disease gene prediction. Brief. Funct. Genomics, 10, 280–293. 21764832 10.1093/bfgp/elr024

[bty559-B35] Zhou H., Skolnick J. (2016) A knowledge-based approach for predicting genedisease associations. Bioinformatics, 32, 2831–2838. 27283949 10.1093/bioinformatics/btw358 PMC5018378

